# Role of prophylactic mesh in emergency midline laparotomy: a systematic review and meta-analysis

**DOI:** 10.1186/s13017-026-00697-9

**Published:** 2026-04-24

**Authors:** Mahmoud Diaa Hindawi, Abdel-Fattah Kalmoush, Mohamed Gamal Mohamed, Ezzeldin Ahmed Abdelaty, Abdulrahman Faisal Ziada, Waleed Abdelrhman Kotb, Mohamed Mostafa Eisa, Hamdi Elbelkasi, Richard Peter ten Broek, Edward C.T.H. Tan, Andrew W. Kirkpatrick

**Affiliations:** 1 Faculty of Medicine, Al-Azhar University, Cairo, Egypt; 2 General Surgery Department, Al-Azhar University, Cairo, Egypt; 3 Faculty of Medicine, Assiut University, Asyut, Egypt; 4 Faculty of Medicine, Al-Azhar University, Damietta, Egypt; 5 Faculty of Medicine, Tanta University, Tanta, Egypt; 6 Faculty of Medicine, Kafr El-Sheikh University, Kafr El-Sheikh, Egypt; 7 Matariya Teaching Hospital (GOTHI), Cairo, Egypt; 8 Department of Surgery, Radboud University Medical Center, Nijmegen, The Netherlands; 9 Departments of Critical Care Medicine and Surgery, Foothills Medical Centre, Calgary, AB Canada

**Keywords:** Emergency laparotomy, Prophylactic mesh, Wound complications, Acute care surgery, Incisional hernia, Hernia, Seroma

## Abstract

**Purpose:**

The role of prophylactic mesh reinforcement in emergency laparotomy closure remains controversial. While prophylactic mesh may reduce incisional hernia, its use in unstable and contaminated settings raises concerns regarding operative time, seroma development, and wound complications. This meta-analysis of randomized controlled trials (RCTs) evaluated the safety and efficacy of prophylactic mesh versus primary suture closure in emergency midline laparotomy.

**Methods:**

A systematic search was performed for RCTs comparing prophylactic mesh with suture closure in adult patients undergoing emergency midline laparotomy. Primary outcomes were overall wound complications (OWC) and incisional hernia (IH). Secondary outcomes included superficial and deep surgical site infection, wound dehiscence (WD), seroma, hematoma, operative time, postoperative pain, quality of life, hospital and ICU stay, transfusion, and mortality.

**Results:**

Seven RCTs comprising 643 patients were included. Mesh reinforcement reduced incisional hernia incidence, with significant reductions at 1 month (RR 0.29, 95% CI 0.12–0.68), 6 months (RR 0.11, 95% CI 0.01–0.86), 12 months (RR 0.21, 95% CI 0.09–0.49), and 24 months (RR 0.27, 95% CI 0.15–0.49). Mesh increased seroma risk (RR 2.45, 95% CI 1.38–4.35) and, was associated with higher overall wound complications (RR 1.50, 95% CI 1.04–2.18). No significant differences were found in SSI, wound dehiscence, hematoma, transfusion, ICU or hospital stay, pain, quality of life, or mortality. Operative time was longer with mesh (MD 26 min, 95% CI 15.9–36.9).

**Conclusion:**

Prophylactic mesh in emergency laparotomy closure poses a clinical dilemma: it lowers the risk of incisional hernia but prolongs surgery and increases seroma and wound complications. Current evidence underscores the trade-off between long-term prevention and short-term morbidity. Larger, protocol-driven trials with long-term follow-up are needed to determine in which patients and wound classes mesh reinforcement is justified.

**Supplementary Information:**

The online version contains supplementary material available at 10.1186/s13017-026-00697-9.

## Introduction

Despite the widespread adoption of modern fascial closure techniques in emergency laparotomy, including continuous suturing with a small-bite approach and slowly absorbable monofilament sutures, there remains a significant gap in definitive evidence regarding the role of prophylactic mesh augmentation [1, 2]. Despite well-established principles of abdominal wall closure, including the use of continuous small-bite sutures with a suture length–to–wound length ratio of at least 4:1, the selection of slowly absorbable monofilament materials, careful attention to wound contamination, and the consideration of adjunctive measures such as negative pressure therapy, emergencies often necessitate a rapid midline laparotomy [2, 3]. The midline incision, while providing the fastest and broadest access, carries a particularly high risk of wound-related complications, most notably wound dehiscence (WD) and incisional hernia (IH) [2, 4].

Within this context, the 2023 World Society of Emergency Surgery (WSES) ECLAPTE consensus acknowledged that prophylactic mesh augmentation may reduce these complications but did not reach sufficient agreement to issue a formal recommendation; instead, it suggested its selective use in high-risk patients and refrained from specifying the mesh type or placement [2]. More recently, the 2024 RCT-only meta-analysis by Marcolin et al. demonstrated that mesh reinforcement significantly reduces the incidence of IH and, when synthetic materials are used, also decreases WD, without increasing infection or reoperation, although at the cost of longer operative times and higher seroma rates [5]. Taken together, these findings highlight the promise of prophylactic mesh in mitigating midline laparotomy complications, while also underscoring the persisting uncertainty that necessitates further high-quality evidence.

Although minimally invasive laparoscopic methods are increasingly used in emergency abdominal surgery, the traditional midline laparotomy remains the most common approach because it provides rapid access to the abdominal cavity and can be easily extended when needed [6]. However, this incision carries a high risk of IH, one of the most frequent and clinically significant complications after abdominal surgery. The risk is particularly elevated in emergency settings and high-risk patients, with IH incidence ranging from 5% to 25% in the general population to as high as 66% in emergency cases [5, 7–9]. IH is not merely a cosmetic concern; it causes pain, abdominal deformity, rehospitalization, and often requires reoperation. Despite advances in suture materials, mesh technology, and closure techniques, recurrence rates remain substantial, with some studies reporting rates of up to 54% [10]. Importantly, previous ventral hernia repair is one of the strongest risk factors for recurrence, creating a vicious cycle of repair, complications, reoperation, and re-repair [11]. Long-term outcomes after ventral hernia repair therefore remain poor, underscoring the need for preventive strategies and improved approaches to durable abdominal wall reconstruction.

Evidence from elective surgery demonstrates that prophylactic mesh reinforcement is effective in preventing IH. For instance, the PRIMA trial showed a markedly lower incidence of hernia in patients treated with mesh (13%) compared with those closed with sutures alone (31%) [12]. More recently, a 2025 systematic review and meta-analysis of 15 randomized trials confirmed that mesh reinforcement significantly reduces IH and the need for reoperation in elective abdominal surgery, particularly in high-risk populations such as patients undergoing bariatric, open abdominal aortic aneurysm, and other gastro-intestinal surgeries, although it is associated with an increased risk of seroma and, to a lesser extent, wound infection. In contrast, evidence in the emergency setting remains limited [13]. As a result, recent systematic reviews and major guidelines, including those of the European and American Hernia Societies, have concluded that current evidence is insufficient and too heterogeneous to recommend routine mesh use in emergency midline laparotomies [2, 12–15].

Here, we aim to update and build upon the limitations of previous literature by incorporating the most recent evidence, addressing some of the existing uncertainties, and highlighting outcomes across a wide range of clinical settings reported in the available trials. In doing so, our study seeks to provide a more comprehensive synthesis that reflects both the progress and the persisting gaps in the role of prophylactic mesh reinforcement during emergency midline laparotomy closure.

## Methods

We adhered to the standards of the Preferred Reporting Items for Systematic Reviews and Meta-Analyses (PRISMA) 2020 statement in the conduct and reporting of this systematic review and meta-analysis [16]. All methodological steps were carried out in accordance with the recommendations outlined in the Cochrane Handbook for Systematic Reviews of Interventions, ensuring rigor and transparency throughout the process [17]. The study protocol was prospectively registered; https://www.crd.york.ac.uk/PROSPERO/view/CRD420251142229.

###  Search strategy and data sources

We performed a systematic literature search in PubMed (Ovid MEDLINE), Embase, Cochrane Central Register of Controlled Trials (CENTRAL), Scopus, and Web of Science to identify relevant studies published from inception to June 12, 2025. The search strategy combined controlled vocabulary and free-text terms, tailored for each database. The following string was applied: (emergency laparotomy OR emergency surgery OR acute abdomen OR emergency abdominal surgery OR emergency midline laparotomy OR Damage control laparotomy) AND (mesh OR surgical mesh OR prosthetic mesh OR prophylactic mesh OR abdominal wall reinforcement OR mesh positioning) NOT (elective OR elective surgery OR elective laparotomy OR elective abdominal surgery OR elective procedures). The detailed search strategy for each database is shown in Electronic Supplementary 1 A (ESM 1 A).

###  Selection criteria

We included randomized controlled trials (RCTs) that investigated prophylactic mesh reinforcement in adult patients undergoing emergency midline laparotomy. Eligible studies primarily compared abdominal fascial closure with mesh augmentation versus closure without mesh. Conventional closure was defined as fascial approximation with continuous or interrupted sutures using absorbable or non-absorbable monofilaments (e.g., polypropylene, polyglactin, or polydioxanone), without additional reinforcement.

Studies were considered eligible if they met the following PICOS framework:

- *Population (P)*: Adults aged > 18 undergoing emergency midline laparotomy.
- *Intervention (I)*: Abdominal fascial closure with prophylactic mesh reinforcement.
- *Comparator (C)*: Fascial closure with sutures alone (no mesh).
- *Outcomes (O)*: Incidence of incisional hernia and wound-related complications, including evisceration, superficial and deep surgical-site infection, seroma, hematoma, operative time, postoperative pain, and quality of life.
- *Study design (S)*: RCTs published in English.

### Exclusion criteria

At the title and abstract screening stage, we excluded studies that involved elective laparotomies only, as well as non-randomized or observational studies, reviews, case reports, case series, animal studies, non-English publications, letters to the editor, conference abstracts, editorials, commentaries, and unpublished works (e.g., dissertations or theses). During full-text screening, studies that included both elective and emergency laparotomies were only eligible if subgroup data for emergency patients were available. Trials involving pregnant participants were also excluded.

###  Outcomes of interest included

The primary outcome of interest was the incidence of incisional hernia, reported at the longest available follow-up and, when possible, at predefined time points (1, 2, 3, 6, 12, and 24 months). Secondary outcomes included overall wound complications, surgical-site infection, classified as superficial or deep fascial dehiscence, seroma, hematoma, operative time measured in minutes, length of intensive care unit stay, and hospital stay measured in days, postoperative pain assessed by visual analogue scale, and quality of life when available. Tertiary outcomes comprised the need for blood transfusion and mortality, whether in-hospital or at the longest follow-up reported.

### Study selection and data extraction

Screening was conducted in two phases. In the first phase, titles and abstracts of all retrieved records were independently screened by three reviewer pairs. In the second phase, the full texts of potentially eligible studies were assessed independently by the same reviewer pairs using the predefined inclusion and exclusion criteria. Any disagreements within a pair were discussed to reach consensus; unresolved conflicts were adjudicated by a third author.

Data extraction was subsequently performed independently by the same three reviewer pairs using a standardized form. Extracted data included study design, setting, follow-up duration, inclusion and exclusion criteria, mesh placement site, mesh type, mesh overlap, fixation technique, drain placement, fascial suture technique, comparator technique, and antibiotic use. Baseline patient characteristics were also extracted, including age, sex, body mass index (BMI), diabetes, chronic obstructive pulmonary disease (COPD), smoking status, prior abdominal surgery, ASA class, and wound classification (clean, clean-contaminated, contaminated, or dirty). Outcomes were then extracted according to the predefined framework: incisional hernia as the primary outcome, with intraoperative, postoperative, and tertiary outcomes as specified. All extracted data were cross-checked among reviewer groups, and discrepancies were resolved by consensus or, when necessary, by a third author.

### Quality assessment

The risk of bias of included trials was assessed independently by two blinded reviewers using the ROB 2 tool [18], which evaluates five domains: randomization process, deviations from intended interventions, missing outcome data, measurement of outcomes, and selection of reported results. Each domain was judged as low risk, some concerns, or high risk. Disagreements were resolved through discussion with a third senior reviewer.

### Data synthesis and statistical analysis

All analyses were performed using R software (version 2024.04.2) with the *meta* package (version 7.0–0) [19]. Effect estimates were expressed as risk ratios (RRs) for dichotomous outcomes and mean differences (MDs) for continuous outcomes, with corresponding 95% confidence intervals (CIs). A random-effects model (DerSimonian and Laird method) was applied to all outcomes to account for between-study variability. Heterogeneity was assessed visually with forest plots and quantitatively using the Cochrane Q test (Chi-square) and the I^2^ statistic. A *p*-value < 0.1 on the Q test indicated significant heterogeneity, while I^2^ values of < 25%, 25–75%, and > 75% were interpreted as low, moderate, and high heterogeneity, respectively [20]. Where heterogeneity was present, results continued to be synthesized under the random-effects model. Prespecified subgroup analyses were conducted primarily according to mesh position and length of follow-up, with particular attention to complications such as hematoma, seroma, and wound infection. Sensitivity analyses were performed using a leave-one-out approach to assess the robustness of pooled estimates.

Sensitivity analyses were performed using a leave-one-out approach to assess the robustness of pooled estimates, prioritizing the exclusion of studies according to their clinical relevance (e.g., differences in patient population, contamination status, or mesh type/placement) before purely statistical influence on heterogeneity.

During the quality assessment, studies judged to have a high risk of bias were reviewed but not included in the quantitative synthesis. This approach was chosen to ensure that our pooled results reflect the most reliable evidence and are not influenced by methodologically weak trials.

### GRADE assessment

We assessed the certainty of evidence for our primary outcomes using the Grading of Recommendations Assessment, Development, and Evaluation (GRADE) framework. The quality of evidence was evaluated for the incidence of overall wound complications, superficial surgical site infection, deep surgical site infection, wound dehiscence, seroma, hematoma, and incisional hernia at all available follow-up time points [21].

## Results

### Study selection and characteristics

**Fig. 1:**
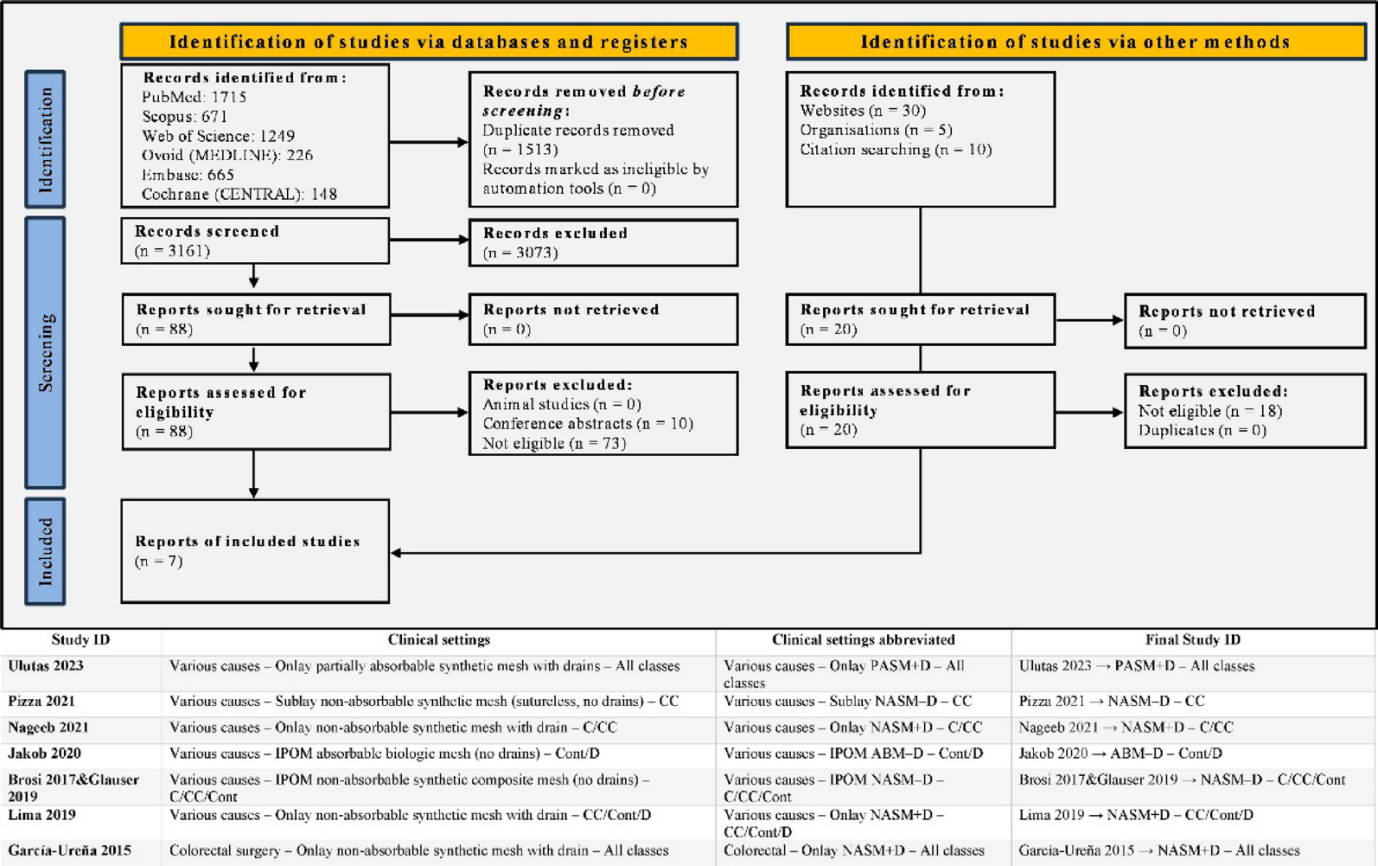
The search strategy flowchart and the included studies’ ID

Seven RCTs published [4, 8, 22–27] between 2015 and 2023 were included, enrolling 643 patients from Turkey, Italy, Egypt, Switzerland, Brazil, and Spain, Fig. 1. Prior to submission, we also performed a manual literature search and screened Google Scholar alerts for any newly published studies. All compared prophylactic mesh reinforcement with standard fascial closure, using onlay (4 trials) [4, 8, 22, 27], sublay (1 trial) [23], or Intraperitoneal Onlay Mesh (IPOM) (2 trials) [24–26] techniques. Mesh types varied from lightweight polypropylene to biosynthetic and biologic prostheses, with drains routinely used in onlay but not in IPOM approaches. Summary and Baseline demographics, as well as comorbidities, are summarized in Tables 1 and 2.

**Table 1:** Summary table

Study ID	Design	Country data-source setting	Duration	Inclusion criteria exclusion criteria	Mesh placement site	Mesh type detailed	Mesh overlap	Mesh suture fixation type	Drain placement	Fascial suture	Comparator technique, suture	Antibiotic details mesh versus non mesh
Ulutas 2023 [22]	Prospective randomized double-blinded study	Turkey Prospectively collected data from patients enrolled in the trial General Surgery Department, University of Health Sciences, Konya City Hospital	August 1, 2020–August 1, 2021, with a minimum follow-up of 12 months	Inclusion criteria Age over 18, emergency midline laparotomy, and having at least two defined risk factors for incisional hernia Exclusion criteria Age under 18, elective operations, non-midline incisions, pre-existing hernia or diastasis recti, laparoscopic operations, metastatic cancer, short lifespan expectation (< 2 years), pregnancy, reproductive age group (15–49), and having less than two risk factors	Onlay (supra-aponeurotic)	Polypropylene, partly absorbable, light, and macroporous (Ultrapro, Ethicon, NJ, USA)	The mesh extended up to 5 cm from the fascia in lateral and craniocaudal positions. A mesh 10 cm longer than the incision and 10 cm wide was used	Fixed at 4 sides with USP 2 − 0 Prolene (polypropylene) sutures, then fixed circumferentially with polypropylene sutures	Subcutaneous negative pressure drainage catheters were used on all patients with mesh	Closed with an easy-absorbable suture (USP 2−0 PDS Plus II) as a linea alba, with a suture length to wound length ratio (SL/WL) of at least 4:1	Conventional abdominal closure Fascial suture closed with an easy-absorbable suture (USP 2 −0 PDS Plus II) as a linea alba, with a suture length to wound length ratio (SL/WL) of at least 4:1 subcutaneous tissue closed with 3/0 polyglactin, and skin closed with staplers	Both groups received cefazolin and metronidazole for prophylaxis
Pizza 2021 [23]	Prospective randomized controlled double-blind trial	Italy Prospectively collected data Department of General and Emergency Surgery of the “A. Rizzoli” Hospital	5 Years from January 2014 to December 2019	Inclusion criteria Patients undergoing an open emergency surgical operation associated with a permanent end colostomy Exclusion criteria Age < 18 years; life expectancy < 12 months; pregnancy; immunosuppressant therapy within 2 weeks before surgery	Sublay	Biosynthetic, absorbable polyglycolide-trimethylene carbonate copolymer (GORE^®^ BIO-A^®^)	A space of at least 8 × 8 cm was created for the mesh The mesh itself measured at least 8 × 8 cm in diameter	The mesh was fixed with four stitches to both the colon and the anterior rectal sheath	NA	Mesh Group Posterior sheath was incised to place the mesh Non-Mesh Group Anterior and posterior sheaths were incised for the stoma	Conventional end-colostomy: A circular skin incision was made, the anterior and posterior sheaths of the rectus muscle were incised, and the colon was brought out transrectally. Four reabsorbable stitches were used for fixation, and mucocutaneous sutures fixed the stoma to the skin	Received routine antibiotic treatment with sulbactam/ampicillin 1.5 g i.v. three times per day for 5 days
Nageeb 2021 [8]	Prospective randomized comparative study	Egypt, Ain Shams University Hospitals Emergency general surgery, single center	January 2017–April 2020, follow-up 24 months	Inclusion criteria Adults undergoing emergency midline laparotomy (clean or clean–contaminated). High-risk patients included (BMI ≥ 30, COPD, DM, malnutrition, and previous midline incision). Exclusion criteria Contaminated/dirty procedures (peritonitis, abscess, perforation), pregnancy, < 18 years, metastatic tumors (short life expectancy), ASA IV–V.	Onlay (supra-aponeurotic, over anterior rectus sheath)	Wide-pore polypropylene (Prolene) mesh, non-absorbable	At least 5 cm dissection lateral to the incision; mesh fixed flush over the sheath	Interrupted 2 − 0 Prolene sutures circumferentially to the sheath edge	Closed-system suction drain (Redivac) placed over mesh	Polydioxanone loop #1, antibacterial coated, continuous technique, 1 cm bite/1 cm interval, SL: WL ≥ 4:1	Standard continuous closure with the same PDS loop suture, SL: WL ≥ 4:1	Prophylaxis with third-generation cephalosporin at induction; wound irrigation with saline + gentamicin in the mesh group
Jakob 2020 [24]	two-arm randomized clinical trial	Switzerland Bern University Hospital	“18 months (planned) The study had to be terminated early.”	Inclusion criteria patients who underwent midline laparotomy or laparoscopy with expected conversion to midline laparotomy for abdominal emergencies Exclusion criteria moribund patients indicated as an American Society of Anesthesiologists physical status classification system score of 5 -patients with septic shock requiring vasoactive medication, pregnancy, prior mesh implantation, or known sensitivity to porcine material or polysorbate 20	IPOM	acellular porcine dermal mesh (Strattice, Allergan)	at least 5 cm in all quadrants	fixed with single stitches using Prolene 2/0 in all four corners the borders of the mesh were fixed using polydioxanon 2/0 running sutures	not reported	Running suture using polydioxanon 1 loops in a 4:1 ratio. The distance of the sutures to the fascial border was 1 cm, and the distance between two stitches was not more than 1 cm	Same continuous fascial closure (PDS loop, 4:1 ratio) without mesh	Not specified (institutional protocol)
Brosi 2017 and Glauser 2019 [25, 26]	monocentric, parallel-group randomized, controlled, open-labelled trial	Switzerland	June 2008–May 2013. 5-year follow-up	Inclusion criteria All patients with a planned median laparotomy. Exclusion criteria - pregnancy- perforation of a hollow viscus- drug addiction - life expectancy less than 5 years due to an advanced tumour stage -known allergy to mesh material- planned second laparotomy – age under 18 years - incapacitated patients. If the patient had an exclusion criterion only found during surgery (i.e., contamination grade IV), they were excluded from randomization	IPOM	7.5 cm wide ParietexTM composite mesh (CovidienTM, Dublin, Ireland) Absorbable, hydrophilic film on the visceral side consisting of porcine collagen, polyethylene glycol and glycerol	The edges of the mesh stripe overlapped the line of the incision by 4 cm, each towards the chest and the pubic symphysis	The mesh stripe was grasped by the polydioxanone loop sutures (PDS II size 1; EthiconTM by Johnson & JohnsonTM, New Brunswick, NJ, USA) and fixed in the midline	Subcutaneous drains were not used.	late-absorbable monofilament polydioxanone loop sutures (PDS II size 1; EthiconTM by Johnson & JohnsonTM, New Brunswick, NJ, USA) with a new suture every 10 cm. The stitches were done every centimetre with a distance to the midline of at least one centimetre. with a thread to incision length ratio of 4:1	the same procedure as the interventional group, without the additional IPOM	NR
Lima 2019 [4]	Prospective randomized clinical trial	Brazil, Hospital das Clínicas da Universidade de São Paulo Emergency general surgery, single center	June 2015–Feb 2018; 30-day primary follow-up (long-term follow-up ongoing)	Inclusion criteria Adults undergoing *emergency midline laparotomy* at high risk for fascial dehiscence, defined by the Rotterdam risk model (≥ 4.0 or ≥ 2.2 plus obesity, malnutrition, malignancy, smoking). Exclusion criteria Laparotomy <¼ xyphoid–pubis distance, prior mesh/incisional hernia, repeat laparotomy < 30d, pregnancy, severe hemodynamic instability, need for open abdomen or relaxing incisions, relaparotomy/death within 30 days (unless FD occurred).	Onlay (supra-aponeurotic, anterior rectus sheath)	Polypropylene (pore 0.8 mm, weight 100 g/m^2^, thickness 0.5 mm; Intracorp^®^/Abdotex^®^)	3 cm overlap in all directions; mesh strip 6 cm wide, 6 cm longer than wound length	Multiple running sutures (polyglactin 2/0) peripherally + concentrically to eliminate dead space	Suction drain above mesh, removed when < 50 mL/day	Continuous slowly absorbable monofilament (PDS 0, 36 mm needle), small-bite 5 mm × 5 mm, SL: WL ≥ 4:1 target (achieved in ~ 30%)	Same fascial closure without mesh reinforcement	Not explicitly detailed (institutional emergency protocol; standard prophylaxis applied)
García-Ureña 2015 [27]	Randomized Controlled Trial (RCT)	Spain Department of General Surgery of Henares University Hospital	2 years	Inclusion criteria: Patients older than 18 years who underwent surgery for any colorectal disease (both elective and emergency surgical procedures) through a midline laparotomy since June 2009 were included. Exclusion criteria: Patients with a previous incisional hernia (IH), intraoperative finding of carcinomatosis, or hemodynamic instability during the surgical procedure were excluded	Onlay	A large-pore with a diameter of 3.6 mm, very low-weight polypropylene mesh (Optilene Mesh Elastic; B. Braun, Melsungen, Germany)	overlapping the cranial and caudal edges by 2 and 2.5 cm, respectively, at each side of the closure, and the two strips themselves overlapped by 2 cm at their point of superimposition	interrupted stitches of poly-p-dioxanone 3/0	A closed aspiration drain was placed over the mesh and removed after 72 h	The linea alba was closed with continuous long-term absorbable suture using poly-4-hydroxybutyrate no. 1, following a 4:1 ratio between suture length and laparotomy length. Running sutures were placed only in the aponeurosis, 1 cm apart and 1 cm from the cut edge.	- The abdominal wall was closed by continuous long-term absorbable suture using poly-4-hydroxybutyrate no. 1, following the 4:1 ratio between suture length and laparotomy length. -The linea alba was closed with running sutures placed only in the aponeurosis from the angle of the wound, spaced 1 cm apart and 1 cm from the cut edge. - The subcutaneous tissue was closed by interrupted polyglactin 2/0 stitches. - Staples were used for skin closure.	Superficial surgical site infections (SSIs) both groups were managed with antibiotics and drainage if necessary

Abbreviations: SSO = surgical site occurrence; SSI = surgical site infection; SWD = surgical wound dehiscence; IH = incisional hernia; NR = not reported; NA = not applicable

**Table 2:** Baseline table data shown as mean (± SD) or n (%) unless stated

Study ID	Age (mean ± SD)	Number of patients	Gender male/female	BMI (mean ± SD)	Diabetes	COPD	Smoking	Prior abdominal surgery	ASA Class	Wound classification
Ulutas 2023 [22]	Mesh 54.7 (± 19.9) Non-mesh 57.1 (± 21.3)	Mesh 50 Non-mesh 51	Mesh 34/16 Non-mesh 25/26	Mesh 27.1 ± 4.4 Non-mesh 26.7 ± 4.9	Mesh 10 Non-mesh 13	Mesh 0 Non-mesh 6	Mesh 22 Non-mesh 17	Mesh 8 Non-mesh 9	Mesh I 3, II 27, III 10, IV 10 Non-mesh I 4, II 20, III 23, IV 4	Mesh Clean 12, Clean-contaminated 24, Contaminated 0, Dirty 14 Non-mesh Clean 5, Clean-contaminated 30, Contaminated 1, Dirty 15
Pizza 2021 [23]	Mesh 66.6 (± 8.6) Non-mesh 69.7 (± 9.8)	Mesh 100 Non-mesh 100	Mesh 38/62 Non-mesh 48/52	Mesh 28.8 (19–34) Non-mesh 28.6 (22–34)	Mesh 11 Non-mesh 13	Mesh 8 Non-mesh 13	Mesh 13 Non-mesh 16	Mesh 15 Non-mesh 8	ASA Class (I-II / III-IV) Mesh: 39/16 Non-mesh: 36 / 19	All patients Clean-contaminated (CDC grade II)
Nageeb 2021 [8]	Mesh 40.26 (± 13.67) Non-mesh 43.08 (± 14.03)	Mesh 38 Non-mesh 34	Mesh 29/9 Non-mesh 22/12	Mesh 33.0 (± 5.8) Non-mesh 31.0 (± 5.8)	Mesh 8 Non-mesh 10	Mesh 3 Non-mesh 3	Mesh 5 Non-mesh 2	Mesh 3 Non-mesh 4	Reported as ≤ III (ASA IV–V excluded) → NR per group	Mesh Clean 11; Clean-contaminated 27 Non-mesh Clean 13; Clean-contaminated 21
Jakob 2020 [24]	Mesh 69.0 (± 8.15) Non-mesh 71.0 (± 11.1)	Mesh 21 Non-mesh 27	Mesh 13/8 Non-mesh 15/12	Mesh 25.5 (± 3.1) Non-mesh 25.4 (± 5.9)	Mesh 1 Non-mesh 6	Mesh* 2 Non-mesh 9		Mesh 12 Non-mesh 19	Mesh II 6, III 9, IV 5, V 1 Non-mesh II 6, III 6, IV 13, V 2	Intraop contamination graded by fluid Mesh Clear 13, Purulent 5, Fecal 1 Non-mesh Clear 15, Purulent 7, Fecal 5.
Brosi 2017 and Glauser 2019 [, ]24, 25	Mesh 64.1 (± 13.02) Non-mesh 65.1 (± 11.79 )	Mesh 131 Non-mesh 136	Mesh 60/71 Non-mesh 56/80)	Mesh 25.8 (± 7.4) Non-mesh 26.6 (± 4.7)	Mesh 13 Non-mesh 14	Mesh 43 Non-mesh 42		Mesh 16 Non-mesh 21	NR	Mesh Clean 39 (29.8%), Clean-contaminated 31 (23.7%), Contaminated 61 (46.6%), Dirty excluded Non-mesh Clean 40 (29.4%), Clean-contaminated 32 (23.5%), Contaminated 64 (47.1%), Dirty excluded
Lima 2019 [4]	Mesh 61.0 (± 12.6) Non-mesh 66.1 (± 12.3)	Mesh 63 Non-mesh 52	Mesh 35/28 Non-mesh 31/21	Mesh 26.0 (± 7.3) Non-mesh 24.8 (± 5.0)	Mesh 6 Non-mesh 11	Mesh 4 Non-mesh 2	Mesh 13 Non-mesh 7	Mesh 7 Non-mesh 9	Mesh I 9 (14.5%), II 31 (49.2%), III 22 (34.9%), IV 1 (1.6%) Non-mesh I 9 (17.3%), II 22 (42.3%), III 21 (40.4%), IV 0	Mesh Clean-contaminated 37 (58.7%), Contaminated 10 (15.9%), Dirty 16 (25.4%) Non-mesh Clean-contaminated 29 (55.8%), Contaminated 9 (17.3%), Dirty 14 (26.9%)
García-Ureña 2015 [27]	Mesh 65.6 (± 13.3) Non-mesh 61.46 (± 15.6)	Mesh 53 Non-mesh 54	Mesh 31/22 Non-mesh 33/21	Mesh 24 Non-mesh 22	Mesh 18 Non-mesh 9	NR	Mesh 5 Non-mesh 9	Mesh 8 Non-mesh 13	NR	Mesh Clean 1 (1.9%), Clean-contaminated 45 (84.9%), Contaminated 5 (9.4%), Dirty 2 (3.8%) Non-mesh Clean 0, Clean-contaminated 42 (77.8%), Contaminated 6 (11.1%), Dirty 6 (11.1%)

### Quality of included studies

Risk of bias across the included RCTs is summarized in ESM 1B. Overall, three trials were judged at low risk, three had some concerns, and one (Brosi & Glauser) was at high risk due to missing outcome data. The most frequent issues were deviations from intended interventions, incomplete outcome reporting, and inconsistent definitions of wound-related endpoints.

### Meta-analysis

#### Primary outcomes

*Incisional hernia (IH)*

Our main analysis was stratified by follow-up period. At 1 month (one RCT, 200 patients), 6/100 mesh patients and 21/100 suture patients developed IH, showing a significant reduction with mesh reinforcement (RR = 0.29, 95% CI [0.12–0.68]). At 3 months (one RCT, 200 patients), 0/100 mesh patients and 2/100 suture patients developed IH, with no significant difference (RR = 0.20, 95% CI [0.01–4.11]). At 6 months (one RCT, 200 patients), 1/100 mesh patients and 9/100 suture patients developed IH, again favoring mesh (RR = 0.11, 95% CI [0.01–0.86]).

At 12 months (two RCTs, 301 patients), 6/150 mesh patients and 29/151 suture patients developed IH. The pooled analysis demonstrated a significant reduction in IH with mesh (RR = 0.21, 95% CI [0.09–0.49], I^2^ = 0%).

At 24 months (three RCTs, 379 patients), 13/191 mesh patients and 47/188 suture patients developed IH. The pooled analysis again showed a significant reduction with mesh (RR = 0.27, 95% CI [0.15–0.49], I^2^ = 0%).

**Fig. 2:**
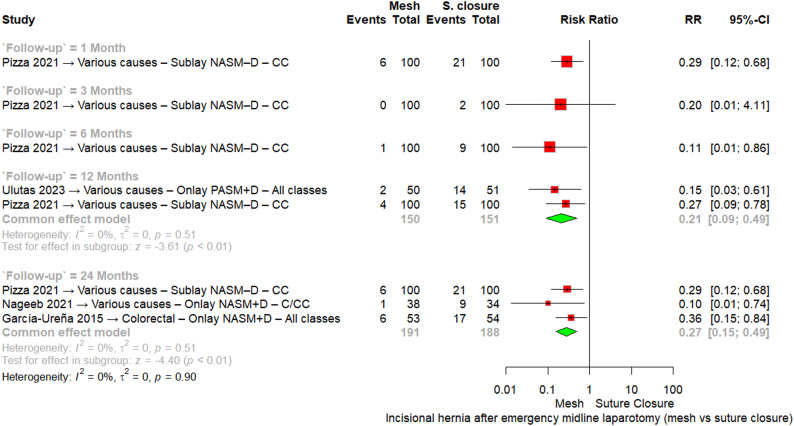
Meta-analysis forest plot of incisional hernia after emergency midline laparotomy at different follow-up intervals. Abbreviations: RR = risk ratio; CI = confidence interval; CC = clean-contaminated; C = clean; Cont = contaminated; D = dirty; NASM–D = non-absorbable synthetic mesh with drain; PASM+D = partially absorbable synthetic mesh with drain

Across all time points, mesh reinforcement was consistently associated with a lower incidence of IH after emergency midline laparotomy, without evidence of heterogeneity, Fig. 2.

*Overall wound complications (OWC)*

Our main analysis was performed by mesh position using a fixed-effect model. In the onlay subgroup (four RCTs, 395 patients), 81/204 mesh patients and 83/191 suture patients developed wound complications, with no significant difference between groups (RR = 1.19, 95% CI [0.91–1.55], I^2^ = 72%). In the sublay subgroup (one RCT, 200 patients), 13/100 mesh patients and 9/100 suture patients were affected (RR = 1.44, 95% CI [0.65–3.23]). In the IPOM subgroup (one RCT, 48 patients), 4/21 mesh patients and 2/27 suture patients developed complications (RR = 2.57, 95% CI [0.52–12.72]). When all six trials were pooled, 98/325 mesh patients and 94/318 suture patients developed OWC, with no significant difference overall (RR = 1.25, 95% CI [0.98–1.61], *p* = 0.08; I^2^ = 58%) ESM. 2 A.

Using a random-effects model, the results remained nonsignificant (RR = 1.29, 95% CI [0.85–1.97], *p* = 0.23; I^2^ = 58%) ESM. 2B.

**Fig. 3:**
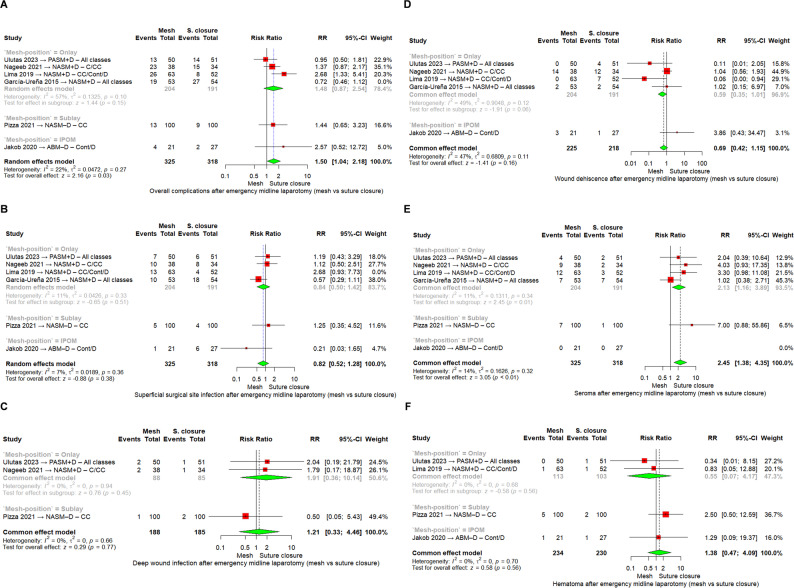
Meta-analysis forest plots of (**A**) Overall complications, (**B**) superficial surgical site infection, (**C**) deep wound infection, (D) wound dehiscence, (**E**) seroma, (**F**) hematoma. Abbreviations: RR = risk ratio; CI = confidence interval; CC = clean-contaminated; C = clean; Cont = contaminated; D = dirty; NASM–D = non-absorbable synthetic mesh with drain; PASM+D = partially absorbable synthetic mesh with drain; ABM–D = absorbable biologic mesh with drain; IPOM = intraperitoneal onlay mesh

In the sensitivity analysis excluding García-Ureña 2015 [27], subgroup analysis by mesh position showed that results for onlay (three RCTs, 342 patients: 62/151 mesh vs. 37/138 suture remained nonsignificant, Fig. 3 A.

However, when all mesh positions were combined after exclusion (five RCTs, 590 patients), 79/272 mesh patients and 48/259 suture patients developed OWC. This pooled random-effects model demonstrated a statistically significant increase in wound complications with mesh reinforcement (RR = 1.50, 95% CI [1.04–2.18], *p* = 0.03; I^2^ = 22%), Fig. 3 A.

*Superficial surgical site infection (SSSI)*

Our main analysis was performed by mesh position using a fixed-effect model. In the onlay subgroup (four RCTs, 395 patients), 40/204 mesh patients and 36/191 suture patients developed superficial SSI, with no significant difference (RR = 1.05, 95% CI [0.70–1.58], I^2^ = 52%). In the sublay subgroup (one RCT, 200 patients), 5/100 mesh patients and 4/100 suture patients were affected (RR = 1.25, 95% CI [0.35–4.52]). In the IPOM subgroup (one RCT, 48 patients), 1/21 mesh patients and 6/27 suture patients developed superficial SSI (RR = 0.21, 95% CI [0.03–1.65]) ESM. 3 A, B.

In the sensitivity analysis excluding Lima 2019 [4], subgroup analysis by mesh position again showed no significant effect: onlay subgroup (three RCTs, 332 patients: 27/151 mesh vs. 24/138 suture, RR = 0.84, 95% CI [0.50–1.42], I^2^ = 11%), Fig. 3B.

When these five RCTs were pooled (580 patients), 33/272 mesh patients and 34/259 suture patients developed superficial SSI. The random-effects model showed no significant difference between groups (RR = 0.82, 95% CI [0.52–1.28], *p* = 0.38; I^2^ = 7%), Fig. 3B.

*Deep surgical site infection (DSSI)*

Our main analysis was performed by mesh position using a fixed-effect model. In the onlay subgroup (two RCTs, 173 patients), 4/88 mesh patients and 2/85 suture patients developed deep SSI, with no significant difference (RR = 1.91, 95% CI [0.36–10.14], I^2^ = 0%). In the sublay subgroup (one RCT, 200 patients), 1/100 mesh patients and 2/100 suture patients were affected (RR = 0.50, 95% CI [0.05–5.43], I^2^ = 0%), Fig. 3C.

When all three RCTs were pooled (373 patients), 5/188 mesh patients and 4/185 suture patients developed deep SSI. The fixed-effect model showed no significant difference between groups (RR = 1.21, 95% CI [0.33–4.46], *p* = 0.77; I^2^ = 0%), Fig. 3C.

*Wound dehiscence*

Our main analysis was performed by mesh position using a fixed-effect model. In the onlay subgroup (four RCTs, 395 patients), 16/204 mesh patients and 25/191 suture patients developed wound dehiscence. The pooled analysis showed no significant difference between groups (RR = 0.59, 95% CI [0.35–1.01], I^2^ = 49%). In the IPOM subgroup (one RCT, 48 patients), 3/21 mesh patients and 1/27 suture patients developed wound dehiscence (RR = 3.86, 95% CI [0.43–34.47]), Fig. 3D.

When all five RCTs were pooled (443 patients), 19/225 mesh patients and 26/218 suture patients developed wound dehiscence. The fixed-effect model demonstrated no significant difference between mesh and suture closure (RR = 0.69, 95% CI [0.42–1.15], *p* = 0.16; I^2^ = 47%), Fig. 3D.

*Seroma*

Our main analysis was performed by mesh position using a fixed-effect model. In the onlay subgroup (four RCTs, 395 patients), 32/204 mesh patients and 14/191 suture patients developed seroma. Mesh reinforcement was associated with a significantly increased risk of seroma (RR = 2.13, 95% CI [1.16–3.89], I^2^ = 11%). In the sublay subgroup (one RCT, 200 patients), 7/100 mesh patients and 1/100 suture patient developed seroma (RR = 7.00, 95% CI [0.88–55.86]). In the IPOM subgroup (one RCT, 48 patients), no cases of seroma were reported in either group, Fig. 3E.

When all six RCTs were pooled (643 patients), 39/325 mesh patients and 15/318 suture patients developed seroma. The fixed-effect model demonstrated a statistically significant increase in seroma with mesh reinforcement compared with suture closure (RR = 2.45, 95% CI [1.38–4.35], *p* < 0.01; I^2^ = 14%), Fig. 3E.

*Hematoma*

Our main analysis was performed by mesh position using a fixed-effect model. In the onlay subgroup (two RCTs, 216 patients), 1/113 mesh patients and 2/103 suture patients developed hematoma, with no significant difference (RR = 0.55, 95% CI [0.07–4.17], I^2^ = 0%). In the sublay subgroup (one RCT, 200 patients), 5/100 mesh patients and 2/100 suture patients developed hematoma (RR = 2.50, 95% CI [0.50–12.59]). In the IPOM subgroup (one RCT, 48 patients), 1/21 mesh patients and 1/27 suture patients developed hematoma (RR = 1.29, 95% CI [0.09–19.37]), Fig. 3F.

When all four RCTs were pooled (464 patients), 7/234 mesh patients and 5/230 suture patients developed hematoma. The fixed-effect model demonstrated no significant difference between groups (RR = 1.38, 95% CI [0.47–4.09], *p* = 0.56; I^2^ = 0%), Fig. 3F.

#### Secondary outcomes

*Operation time*

Our main analysis was performed by mesh position using a fixed-effect model. In the onlay subgroup (four RCTs, 395 patients), mesh reinforcement was associated with significantly longer operative time compared with suture closure, with a pooled mean difference of 25.42 min (95% CI [14.49–36.35], I^2^ = 0%). In the IPOM subgroup (one RCT, 48 patients), mesh placement prolonged operative time by 38 min on average, but this result did not reach statistical significance (MD = 38.00, 95% CI [− 0.23 to 76.23]).

**Fig. 4:**
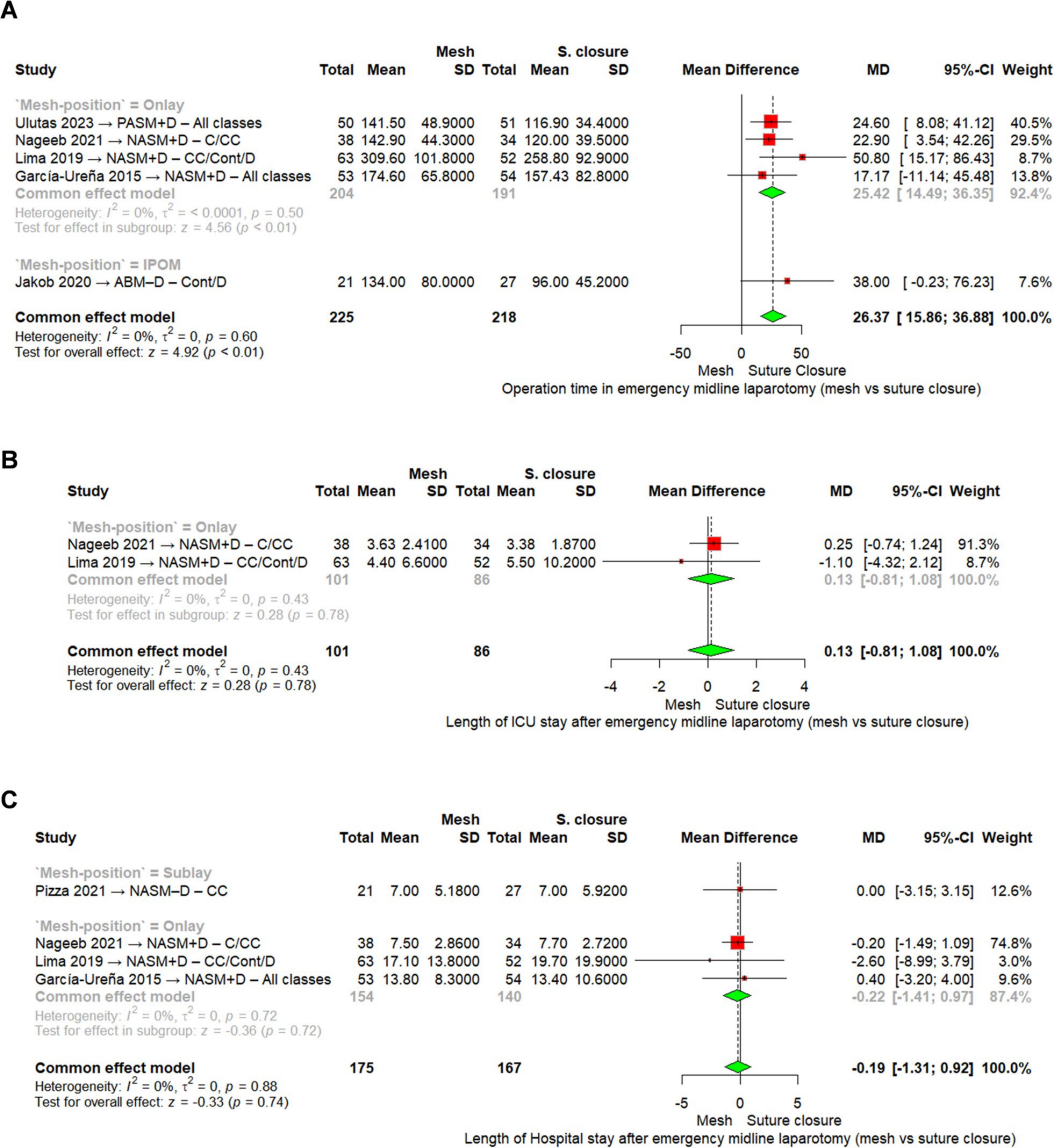
Meta-analysis forest plots of **A** Operation time, **B** length of ICU stay, **C** length of hospital stay. Abbreviations: MD = mean difference; CI = confidence interval; CC = clean-contaminated; C = clean; Cont = contaminated; D = dirty; NASM–D = non-absorbable synthetic mesh with drain; PASM + D = partially absorbable synthetic mesh with drain; ABM–D = absorbable biologic mesh with drain

When all five RCTs were pooled (443 patients), mesh reinforcement significantly increased the duration of surgery by a mean of 26.37 min (95% CI [15.86–36.88], *p* < 0.01; I^2^ = 0%), Fig. 4A.

*Length of ICU stay*

Our main analysis was performed by mesh position using a fixed-effect model. In the onlay subgroup (two RCTs, 187 patients), the mean ICU stay was similar between groups: Nageeb 2021(8) reported a mean difference of 0.25 days (95% CI [− 0.74 to 1.24]), and Lima 2019 [4] reported − 1.10 days (95% CI [− 4.32 to 2.12]). The pooled estimate showed no significant difference (MD = 0.13 days, 95% CI [− 0.81 to 1.08], I^2^ = 0%).

When both studies were combined (187 patients), mesh reinforcement did not significantly affect the length of ICU stay compared with suture closure (MD = 0.13 days, 95% CI [−0.81 to 1.08], *p* = 0.78; I^2^ = 0%), Fig. 4B.

*Length of hospital stay*

Our main analysis was performed by mesh position using a fixed-effect model. In the sublay subgroup (one RCT, 48 patients), there was no difference between groups (MD = 0.00 days, 95% CI [− 3.15 to 3.15]). In the onlay subgroup (three RCTs, 294 patients), hospital stay did not differ significantly: Nageeb 2021(8) (MD = − 0.20 days, 95% CI [− 1.49 to 1.09]), Lima 2019 [4] (MD = − 2.60 days, 95% CI [− 8.99 to 3.79]), and García-Ureña 2015 [27] (MD = 0.40 days, 95% CI [− 3.20 to 4.00]). The pooled onlay analysis showed no difference (MD = − 0.22 days, 95% CI [− 1.41 to 0.97], I^2^ = 0%).

When all four RCTs were pooled (342 patients), mesh reinforcement was not associated with a significant change in hospital stay compared with suture closure (MD = − 0.19 days, 95% CI [−1.31 to 0.92], *p* = 0.74; I^2^ = 0%), Fig. 4C.

*Postoperative pain (VAS score)*

Pain outcomes were reported at multiple follow-up intervals. At the 6th postoperative hour (one RCT, 101 patients), mesh and suture closure had comparable pain scores (MD = − 0.50, 95% CI [− 1.10 to 0.10]). At day 1 (one RCT, 101 patients), no significant difference was observed (MD = − 0.40, 95% CI [− 1.01 to 0.21]). At week 1 (one RCT, 101 patients), pain remained similar between groups (MD = − 0.20, 95% CI [− 0.69 to 0.29]).

At 1 month (two RCTs, 211 patients), pooled results showed no significant difference in pain scores (MD = − 0.10, 95% CI [− 0.61 to 0.41], I^2^ = 2%). At 3 months (one RCT, 101 patients), pain remained comparable (MD = 0.03, 95% CI [− 0.07 to 0.13]). At 6 months (two RCTs, 211 patients), pooled analysis again demonstrated no significant difference (MD = 0.02, 95% CI [− 0.03 to 0.07], I^2^ = 0%).

At 12 months (one RCT, 110 patients), mesh was associated with a marginally lower pain score compared with suture closure (MD = − 0.10, 95% CI [− 0.14 to − 0.06], I^2^ = 68%).

**Fig. 5:**
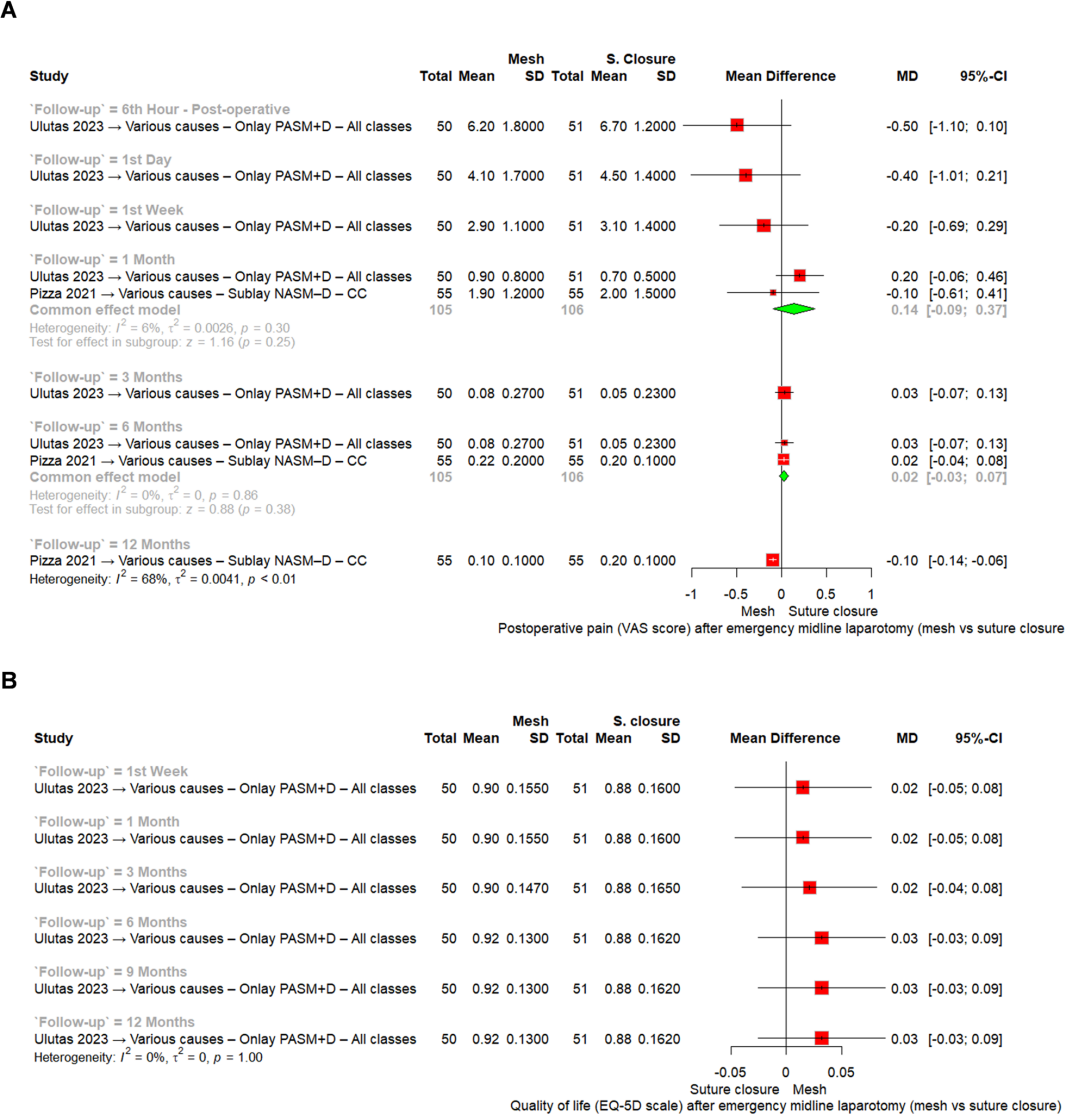
Meta-analysis forest plots of **A** Postoperative pain (VAS score) at different follow-up intervals. **B** Quality of life (EQ-5D scale) at different follow-up intervals. Abbreviations: MD = mean difference; CI = confidence interval; VAS = visual analogue scale; CC = clean-contaminated; C = clean; Cont = contaminated; D = dirty; NASM–D = non-absorbable synthetic mesh with drain; PASM + D = partially absorbable synthetic mesh with drain

Taken together, mesh reinforcement did not meaningfully affect postoperative pain at most time points, with a small reduction only evident at 12 months, Fig. 5A.

*Quality of life (EQ-5D score)*

Quality of life was reported in one RCT (Ulutas 2023 [22], 101 patients) at multiple time points up to 12 months. At 1 week, mean scores were similar between groups (MD = 0.02, 95% CI [− 0.05 to 0.08]). At 1 month (MD = 0.02, 95% CI [− 0.05 to 0.08]), 3 months (MD = 0.02, 95% CI [− 0.04 to 0.08]), 6 months (MD = 0.03, 95% CI [− 0.03 to 0.09]), 9 months (MD = 0.03, 95% CI [− 0.03 to 0.09]), and 12 months (MD = 0.03, 95% CI [− 0.03 to 0.09]), no significant differences were observed.

Overall, mesh reinforcement did not significantly affect postoperative quality of life compared with suture closure across all time points, Fig. 5B.

#### Tertiary outcomes

*Blood transfusion*

Four RCTs (523 patients) reported perioperative blood transfusion. Using a fixed-effect model, 43/266 mesh patients and 50/257 suture patients required transfusion, with no significant difference between groups (RR = 0.82, 95% CI [0.57–1.20], *p* = 0.31), though heterogeneity was substantial (I^2^ = 78%).

When a random-effects model was applied, the result remained nonsignificant (RR = 0.77, 95% CI [0.24–2.53], *p* = 0.67; I^2^ = 78%).

In sensitivity analysis, exclusion of Ulutas 2023 [22] markedly reduced heterogeneity (I^2^ = 0%), yielding a pooled RR of 1.37 (95% CI [0.89–2.11], *p* = 0.15), still showing no significant difference between mesh and suture closure (Fig. 13), ESM 5 A, B, C.

*Mortality*

Three RCTs (270 patients) reported mortality. Pooled analysis demonstrated no significant difference between mesh and primary suture closure (RR 0.79, 95% CI 0.41–1.51, *p* = 0.48), ESM 6.

### Publication bias and quality of evidence

Assessment of publication bias using funnel plots and Egger’s regression test was planned. However, due to the limited number of eligible studies for each outcome, formal testing for publication bias was not feasible [28].

**Fig. 6:**
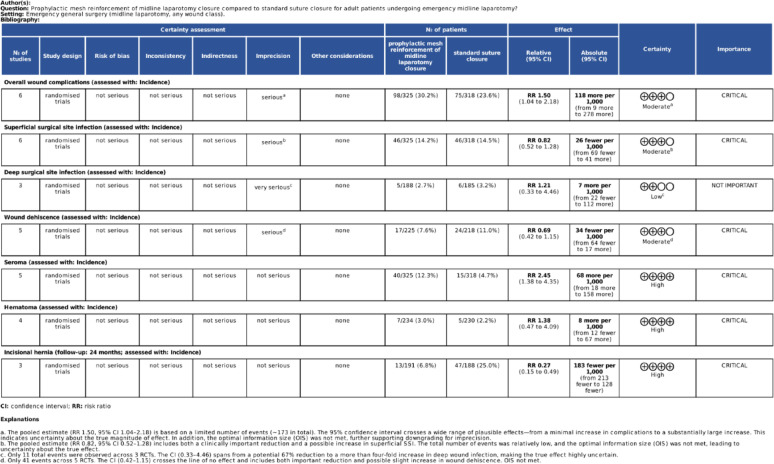
GRADE summary comparing prophylactic mesh versus standard suture closure after emergency midline laparotomy, reporting incidence outcomes with RR and absolute effects and certainty ratings

The GRADE assessment, summarized in Fig. 6, rated the certainty of evidence as high for incisional hernia and seroma, moderate for overall wound complications, superficial SSI, and wound dehiscence, and low for deep SSI. While mesh significantly reduced incisional hernia, it increased seroma and overall wound complications, with no clear differences in SSI, dehiscence, hematoma, systemic morbidity, or mortality.

##  Discussion

Emergency laparotomy remains one of the most common and life-saving procedures in acute care surgery worldwide, particularly in low- and middle-income countries (LMICs), where the burden of emergency abdominal pathology is disproportionately high [29]. Limited access to advanced laparoscopic services, higher prevalence of late presentations, and constrained health system resources make open surgery the default approach for most critically ill patients [30]. Consequently, the morbidity associated with emergency midline laparotomy, including incisional hernia, wound dehiscence, and surgical site infection, represents a significant contributor to postoperative disability, prolonged hospital stays, and increased healthcare costs [31, 32]. In LMICs, where surgical capacity is already strained, these complications compound the problem by consuming scarce hospital resources and adversely affecting patients’ quality of life and productivity [27, 30].

Continuous generation of high-quality evidence in this field is therefore essential. Novel closure techniques and prophylactic strategies, including mesh reinforcement, represent promising methods to reduce postoperative morbidity [5, 31, 33]. However, their applicability in emergency settings, often contaminated, resource-limited, and time-sensitive, remains debated. Our meta-analysis sought to address this gap by synthesizing randomized evidence on prophylactic mesh use in emergency laparotomy, with subgroup analyses by mesh position (onlay, sublay, and IPOM) to provide a more granular assessment of its role in acute care surgery.

In this meta-analysis of seven RCTs including 643 patients undergoing emergency midline laparotomy, prophylactic mesh reinforcement significantly reduced the incidence of incisional hernia at all follow-up points up to 24 months, without heterogeneity. However, mesh was associated with higher rates of seroma and, after sensitivity analysis, an overall increase in wound complications. There were no significant differences in superficial or deep SSI, wound dehiscence, hematoma, blood transfusion, ICU or hospital stay, pain, quality of life, or mortality. Mesh reinforcement consistently prolonged operative time.

The consistent reduction in incisional hernia with mesh is explained by its ability to offload tension from the midline and bridge small fascial gaps that would otherwise heal with weaker scar tissue, a mechanism particularly relevant in emergency laparotomy, where sepsis, malnutrition, and tissue edema impair collagen remodeling. The observed increase in seroma, especially with onlay and sublay placements, reflects the creation of dead space and local inflammatory exudation around the prosthesis, whereas IPOM avoids wide subcutaneous dissection andtherefore showednoseromaformation[34].Recently, closed incision negative pressure wound therapy (ciNPWT) appears to mitigate superficial wound complications, including surgical site infection and wound dehiscence, likely through reduction of edema, exudate, and local inflammatory response, without a significant effect on seroma formation or systemic outcomes. The lack of difference in superficial and deep SSI, wound dehiscence, and hematoma is consistent with the fact that these complications are more strongly influenced by host factors, contamination, and surgical techniqueratherthanthepresenceofprosthesis [35]. In this context, fascial closure technique itself plays a critical role, as the Modified Smead–Jones suture has been shown to reduce wound dehiscence, infection, and hospital stay compared with conventional continuous closure [33].

The rise in overall wound complications after sensitivity analysis was largely driven by seroma, mirroring findings from prior RCT meta-analyse, and indicating that infection-related complications remain unaffected [5, 34]. The longer operative time observed with mesh placement is expected, as additional dissection, mesh sizing, and fixation inevitably add technical steps. Finally, the absence of differences in pain, quality of life, transfusion, ICU or hospital stay, and mortality reflects that these outcomes are primarily determined by patient comorbidities and the severity of the underlying acute condition, rather than by the method of abdominal wall closure. These justifications align with previous syntheses, including Frassini et al. and Marcolin et al., confirming that the main trade-off of mesh reinforcement is more seroma and longer surgery in exchange for a robust and durable reduction in incisional hernia [2, 5, 36].

Incisional hernia is inherently a long-term outcome, and several included trials reported relatively short follow-up periods, particularly at 1 to 6 months. Short follow-up may underestimate the true cumulative incidence of incisional hernia and may not fully capture the durability of prophylactic mesh reinforcement. Although the protective effect of mesh remained evident at 12 and 24 months, the magnitude of benefit may still evolve with longer observation as additional late hernias become clinically apparent. This limitation is particularly important when evaluating preventive interventions, for which long-term follow-up is essential to define the true risk–benefit balance.

Although statistical heterogeneity was low for the primary outcome, the included trials were clinically heterogeneous. Differences in mesh position, prosthetic material, wound class, drain usage, and fascial closure technique may each have modified the observed treatment effects. This underlying variability should be considered when interpreting the pooled estimates.

We observed some inconsistency in the reporting of fascial dehiscence, burst abdomen, and wound evisceration across the included trials. While these entities represent a clinical and anatomical hierarchy, fascial dehiscence being the underlying failure of fascial closure, burst abdomen the acute clinical presentation of fascial ± skin separation, and wound evisceration the most severe manifestation with protrusion of viscera, most studies did not provide a precise definition or distinguish between them. For the purposes of this review, we therefore considered all such events under the unified outcome of fascial dehiscence.

Similarly, reporting of blood transfusion was inconsistent. Only one trial explicitly described postoperative transfusion requirements, while others did not provide data on this outcome. Consequently, results for transfusion were extracted where available but interpreted with caution, as it’s not only not importantly related to our topic, but it’s also important to highlight the inconsistency.

Late complications such as mesh shrinkage, meshoma formation, adhesions, and enterocutaneous fistulae are poorly documented and often manifest many years after implantation [37, 38]. These under-reported events may substantially alter the risk–benefit balance of prophylactic IPOM, especially in resource-limited settings where managing such complications can be catastrophic. True long-term follow-up is therefore essential to fully evaluate the safety profile of this approach.

### Strengths and limitations

This study provides an updated and comprehensive synthesis of randomized evidence on prophylactic mesh reinforcement in emergency midline laparotomy. A key strength lies in our structured subgrouping by mesh position (onlay, sublay, IPOM), which allowed for a nuanced interpretation of anatomical variations in outcomes. We also systematically presented the diversity of clinical settings across trials, including patient populations, wound classifications, and operative contexts, making transparent the heterogeneity that characterizes emergency surgery research. Another strength is the inclusion of outcomes that had been inconsistently reported in the literature, such as quality of life, postoperative pain, ICU stay, hospital stay, and transfusion requirements, thereby broadening the clinical perspective. Sensitivity analyses enhanced the robustness of our conclusions by demonstrating that the observed increase in overall wound complications was mainly attributable to seroma formation rather than infectious complications or fascial failure.

Nevertheless, several limitations must be acknowledged. Definitions of fascial dehiscence, burst abdomen, and evisceration were inconsistently applied across trials, and these entities were therefore analyzed under a unified outcome, which may have introduced misclassification. Reporting of transfusion requirements was heterogeneous, limiting interpretability. While our meta-analysis included six RCTs with over six hundred patients, the absolute number of events for certain outcomes, such as hematoma, deep SSI, and mortality, remained low, reducing statistical power. Follow-up duration varies substantially between studies, which may underestimate long-term complications such as hernia recurrence or chronic pain. Finally, most patients were operated on in clean–contaminated fields, which limits the applicability of our findings to contaminated or dirty emergency laparotomies where the risk–benefit balance of mesh reinforcement may differ.

### Implications for future research

Our analysis raises an important and timely question: Should we deliberately extend operative time in critically ill patients to prevent a complication that may or may not occur in the future? Prophylactic mesh undoubtedly reduces the risk of incisional hernia, but this benefit comes at the cost of longer operations and higher rates of seroma and overall wound complications. In the acute care setting, where patients are physiologically unstable, tissues are often edematous, and contamination risk is higher, every additional minute in the operating room and every potential wound complication may carry meaningful consequences. The burden of incisional hernia is undeniable, but it is also a delayed problem, whereas wound morbidity in the early postoperative period may threaten recovery and survival. This creates a tension between preventing long-term disability and avoiding immediate harm. At the same time, the current literature remains inconsistent and heterogeneous, with trials conducted in widely different clinical settings, wound classifications, and patient populations. As we highlighted, no firm conclusions can yet be drawn about whether prophylactic mesh is appropriate in contaminated, clean, or clean-contaminated fields, nor about which patient profiles stand to benefit most. Therefore, future research should prioritize evaluating mesh reinforcement in clean and clean-contaminated wounds (class I–II), where the biological plausibility of benefit is strongest and safety is more acceptable.

Equally important, upcoming RCTs should adopt strict, protocol-driven methods that leave no detail unspecified. This means:

- Standardized wound closure technique (suture material, size, bite distance, SL: WL ratio).
- Clear antibiotic prophylaxis regimens adapted to wound class.
- Defined patient handling protocols (perioperative glucose control, nutrition, hemodynamic optimization).
- Uniform mesh type, weight, and pore size, with standardized fixation method (corners, continuous vs. interrupted, suture type).
- Explicit instructions on drain placement (site, type, suction vs. gravity) and removal criteria (volume thresholds, time).

Even seemingly minor steps can alter outcomes in this high-risk population, and inconsistent reporting makes evidence synthesis challenging. Only through meticulous protocolization can we determine the true benefit–risk profile of prophylactic mesh in emergency laparotomy.

## Conclusion

Prophylactic mesh reinforcement after emergency midline laparotomy significantly reduces incisional hernia but increases seroma and overall wound complications, while leaving SSI, dehiscence, hematoma, systemic outcomes, and mortality unchanged, at the cost of longer operative time. These findings highlight the trade-off between long-term hernia prevention and short-term wound morbidity in unstable patients. Given the inconsistent clinical settings and wound classifications across existing trials, no solid recommendation can be made regarding which wound classes or patient groups benefit most. High-quality, protocol-driven studies are needed to define when, and for whom, mesh reinforcement should be applied in acute care surgery.

## Supplementary Information

Below is the link to the electronic supplementary material.

Supplementary Material 1

## Data Availability

The data supporting this study are derived from published articles included in the systematic review. All extracted data and the analyses used to generate the findings are available from the corresponding author upon reasonable request.
