# Supplementary material for: Role of prophylactic mesh in emergency midline laparotomy: a systematic review and meta-analysis
Source: World J Emerg Surg. 2026 Apr 24;21:29. doi: 10.1186/s13017-026-00697-9 (PMC13123223; doi:10.1186/s13017-026-00697-9)

Electronic supplementary materials

ESMs

ESM. 1A 2
ESM 1B Quality assessment 3
ESM. 2A - Overall wound complications (OWC) Fixed model 4
ESM. 2B - Overall wound complications (OWC) Random model 4
ESM. 4A Superficial surgical site infection (SSSI) Fixed model 4
ESM. 4B Superficial surgical site infection (SSSI) Random model 5
ESM. 5 A, B, C Blood transfusion 5
A Fixed model 6
B Random model 6
C excluding Ulutas 2023 6
ESM. 6 Mortality 7

ESM. 1A

Databases	Search Strategy for Each Database	
PubMed<br>Ovoid (MEDLINE) (R) ALL<br>Embase<br>Cochrane (CENTRAL)<br>Web of Science	(emergency laparotomy OR emergency surgery OR acute abdomen OR emergency abdominal surgery OR emergency midline laparotomy OR Damage control laparotomy)<br>AND<br>(mesh OR surgical mesh OR prosthetic mesh OR prophylactic mesh OR abdominal wall reinforcement OR mesh positioning)<br>NOT<br>(elective OR elective surgery OR elective laparotomy OR elective abdominal surgery OR elective procedures)	1715<br>226<br>665<br>148<br>1249
Scopus	("emergency laparotomy" OR "emergency surgery" OR "acute abdomen" OR "emergency abdominal surgery" OR "emergency midline laparotomy" OR "Damage control laparotomy")<br>AND<br>("mesh" OR "surgical mesh" OR "prosthetic mesh" OR "prophylactic mesh" OR "abdominal wall reinforcement" OR "mesh positioning")	671

Table 1 shows the search strategy that will be used for each specific database up to June 12, 2025; a total of 4671 after removing duplicates, 3161 were uploaded to Rayyan

ESM 1B Quality assessment

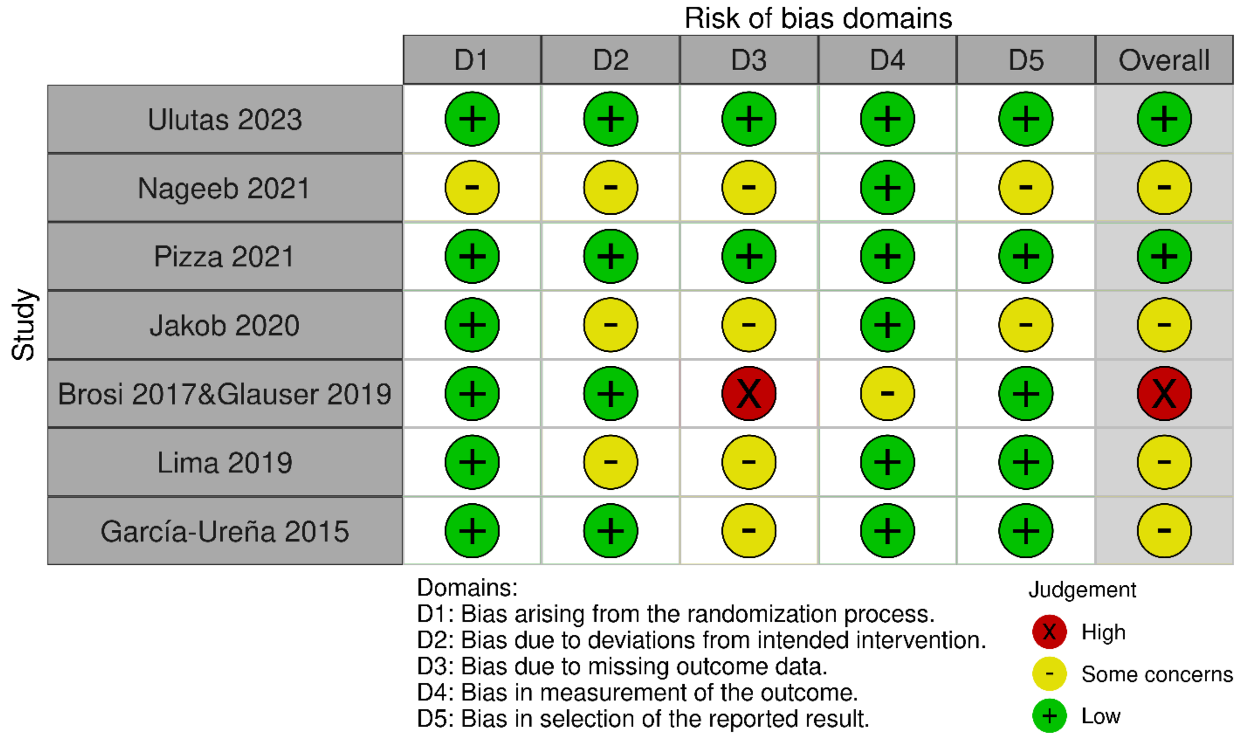

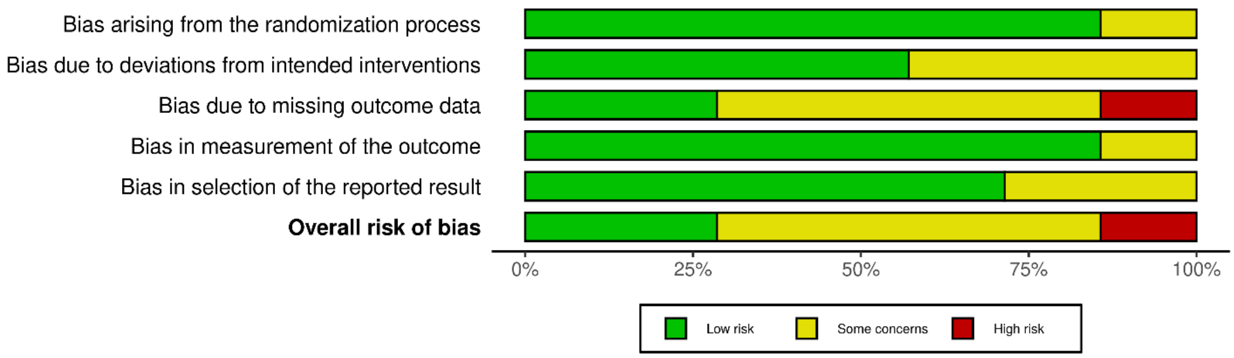

ESM. 2A - Overall wound complications (OWC) Fixed model

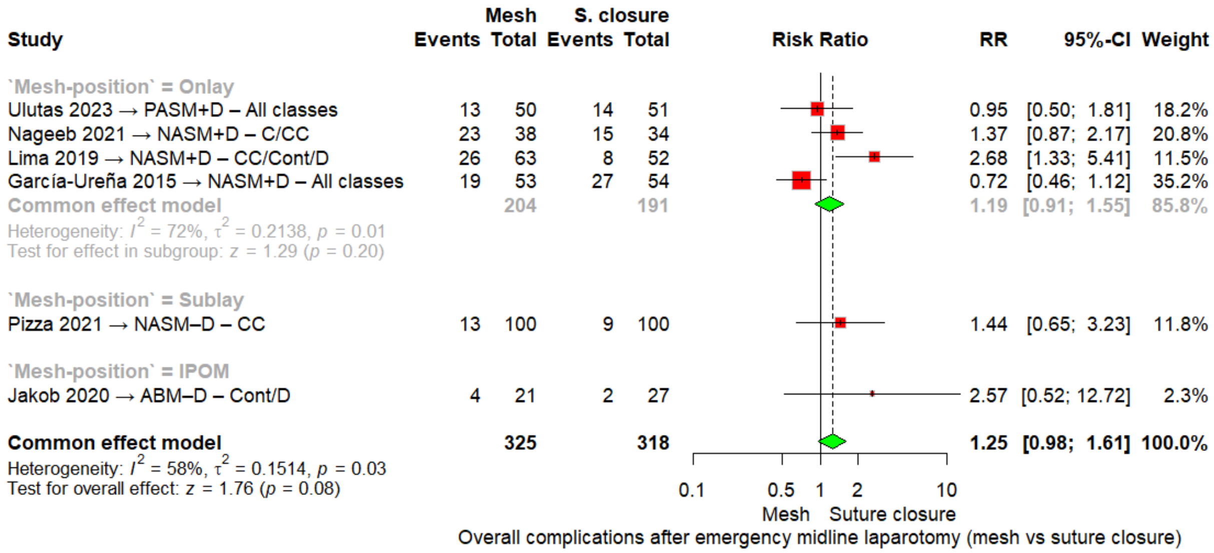

ESM. 2B - Overall wound complications (OWC) Random model

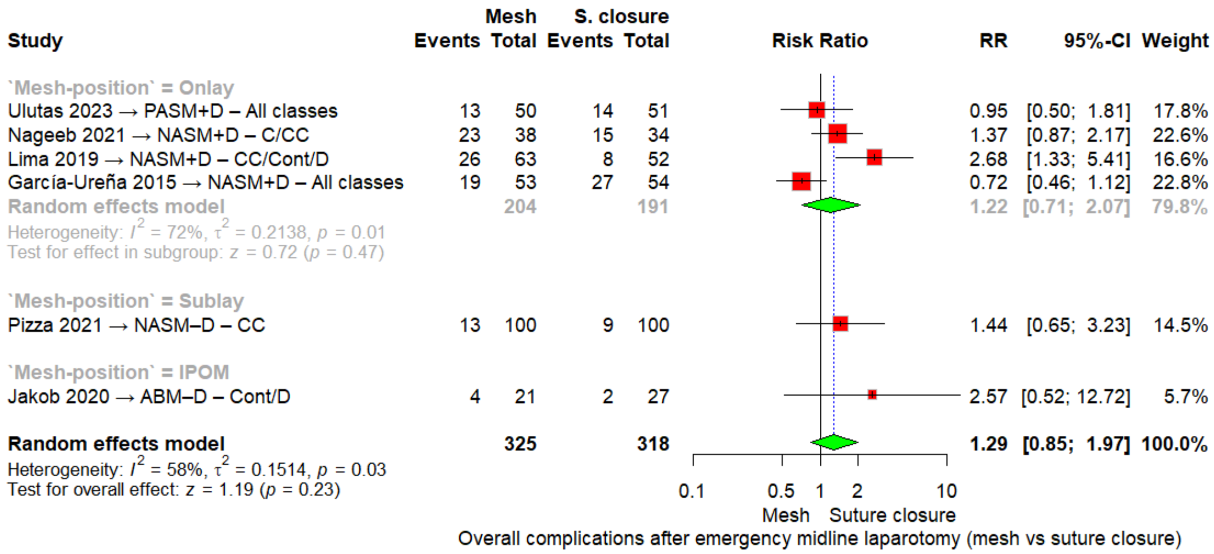

ESM. 3A Superficial surgical site infection (SSSI) Fixed model

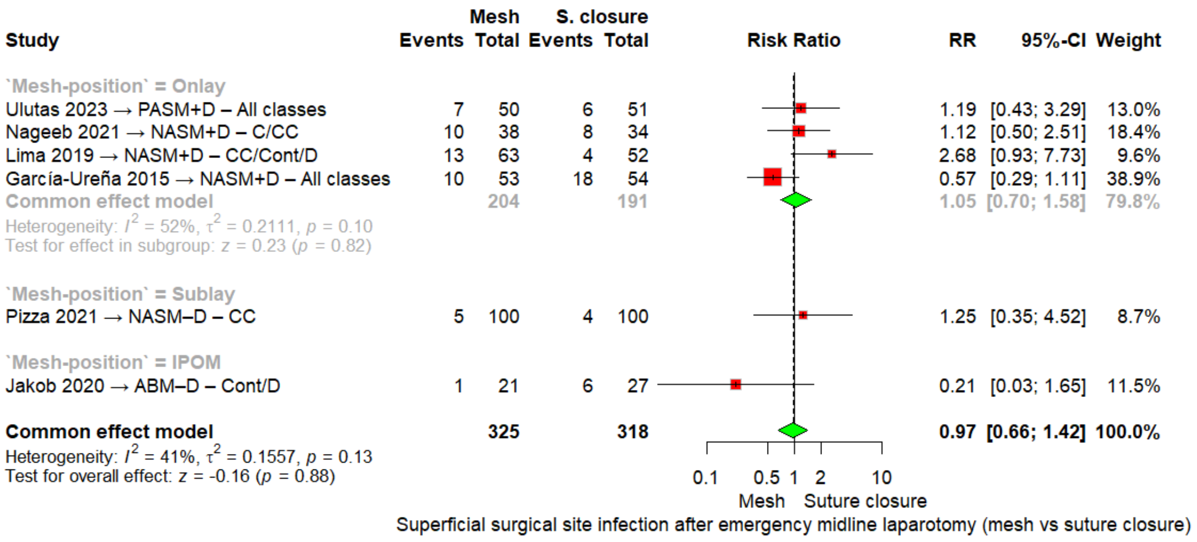

ESM. 3B Superficial surgical site infection (SSSI) Random model

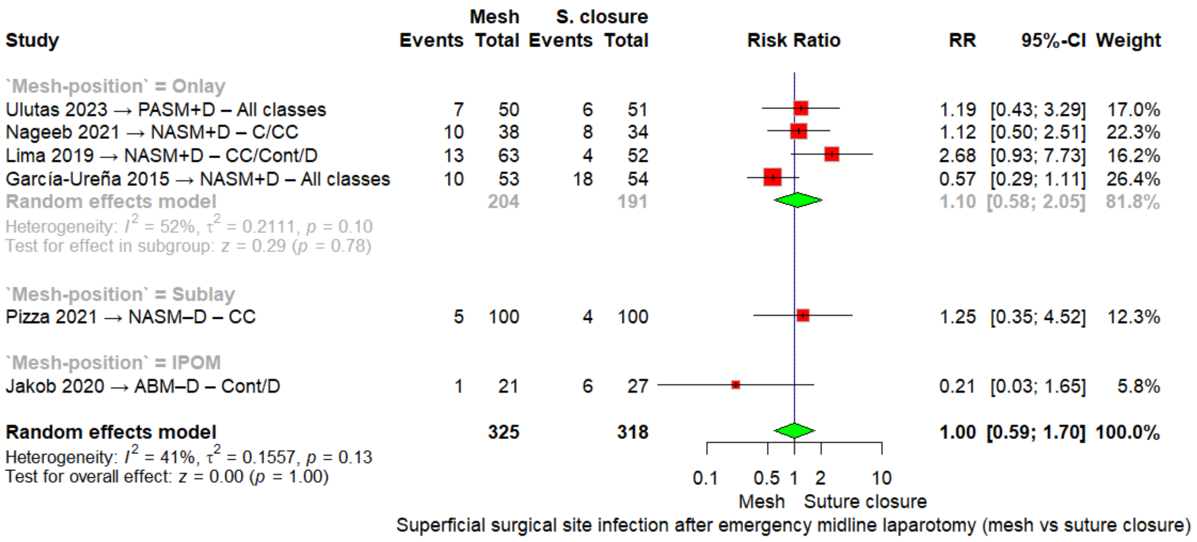

ESM. 4A, B, C Blood transfusion

A Fixed model

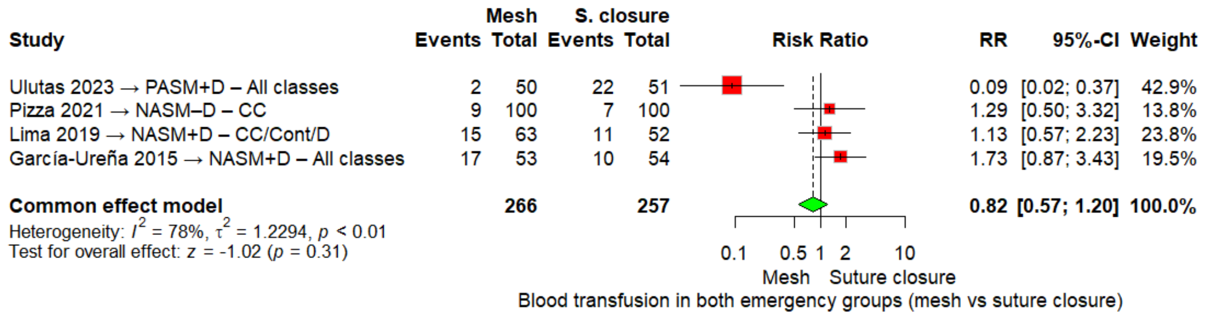

B Random model

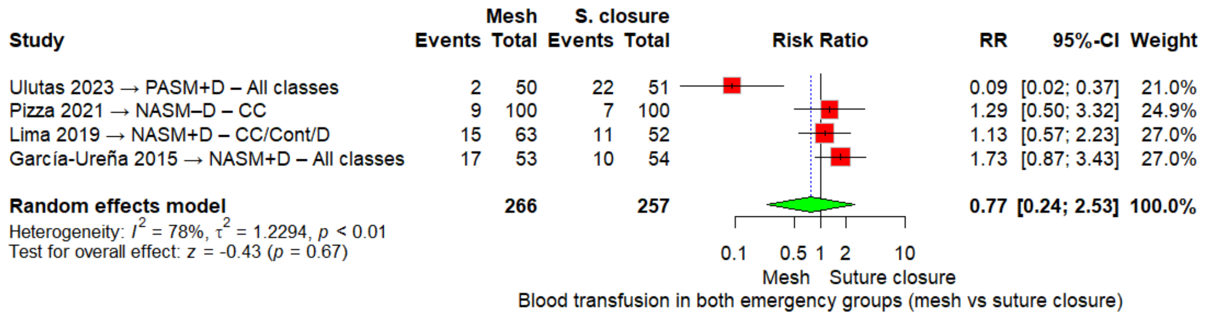

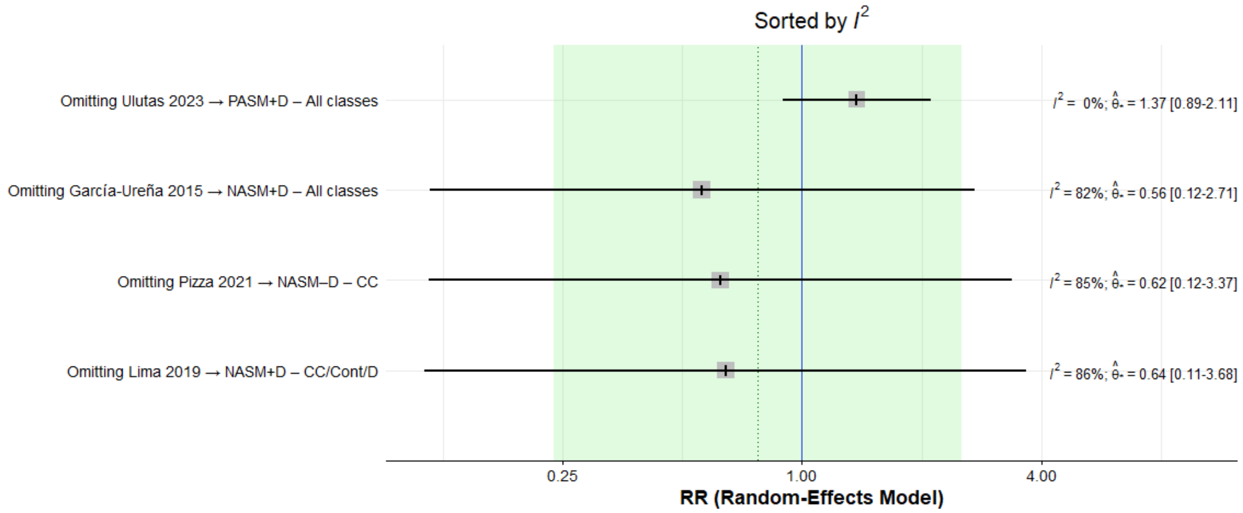

C excluding Ulutas 2023

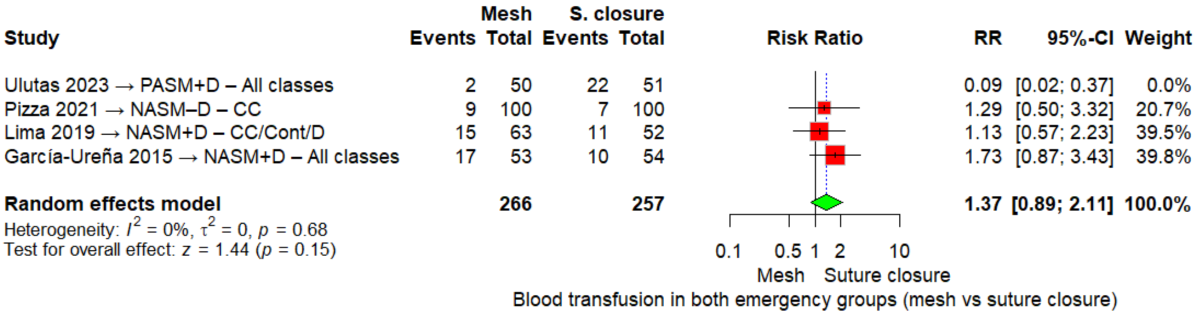

ESM. 5 Mortality

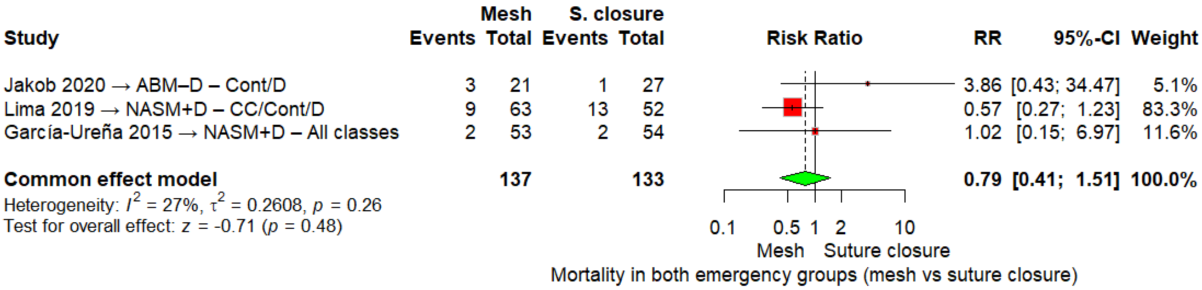

Supplement: Supplementary file 1 — Supplementary Material 1 [file 13017_2026_697_MOESM1_ESM.pdf]
